# The Bacterial Fimbrial Tip Acts as a Mechanical Force Sensor

**DOI:** 10.1371/journal.pbio.1000617

**Published:** 2011-05-10

**Authors:** Pavel Aprikian, Gianluca Interlandi, Brian A. Kidd, Isolde Le Trong, Veronika Tchesnokova, Olga Yakovenko, Matt J. Whitfield, Esther Bullitt, Ronald E. Stenkamp, Wendy E. Thomas, Evgeni V. Sokurenko

**Affiliations:** 1 Department of Microbiology, University of Washington, Seattle, Washington, United States of America; 2 Department of Bioengineering, University of Washington, Seattle, Washington, United States of America; 3 Departments of Biological Structure and Biochemistry, University of Washington, Seattle, Washington, United States of America; 4 Department of Physiology & Biophysics, Boston University School of Medicine, Boston, Massachusetts, United States of America; University of Wisconsin-Madison, United States of America

## Abstract

There is increasing evidence that the catch bond mechanism, where binding becomes stronger under tensile force, is a common property among non-covalent interactions between biological molecules that are exposed to mechanical force in vivo. Here, by using the multi-protein tip complex of the mannose-binding type 1 fimbriae of *Escherichia coli*, we show how the entire quaternary structure of the adhesive organella is adapted to facilitate binding under mechanically dynamic conditions induced by flow. The fimbrial tip mediates shear-dependent adhesion of bacteria to uroepithelial cells and demonstrates force-enhanced interaction with mannose in single molecule force spectroscopy experiments. The mannose-binding, lectin domain of the apex-positioned adhesive protein FimH is docked to the anchoring pilin domain in a distinct hooked manner. The hooked conformation is highly stable in molecular dynamics simulations under no force conditions but permits an easy separation of the domains upon application of an external tensile force, allowing the lectin domain to switch from a low- to a high-affinity state. The conformation between the FimH pilin domain and the following FimG subunit of the tip is open and stable even when tensile force is applied, providing an extended lever arm for the hook unhinging under shear. Finally, the conformation between FimG and FimF subunits is highly flexible even in the absence of tensile force, conferring to the FimH adhesin an exploratory function and high binding rates. The fimbrial tip of type 1 *Escherichia coli* is optimized to have a dual functionality: flexible exploration and force sensing. Comparison to other structures suggests that this property is common in unrelated bacterial and eukaryotic adhesive complexes that must function in dynamic conditions.

## Abstract 2

The subunits that constitute the bacterial adhesive complex located at the tip of the fimbria form a hook-chain that acts as a rapid force-sensitive anchor at high flow.

## Author Summary

Noncovalent biological interactions are commonly subjected to mechanical force, particularly when they are involved in adhesion or cytoskeletal movements. While one might expect mechanical force to break these interactions, some of them form so-called catch bonds that lock on harder under force, like a nanoscale finger-trap. In this study, we show that the catch-bond forming adhesive protein FimH, which is located at the tip of *E. coli* fimbriae, allows bacteria to bind to urinary epithelial cells in a shear-dependent manner; that is, they bind at high but not at low flow. We show that isolated fimbrial tips, consisting of elongated protein complexes with FimH at the apex, reproduce this behavior in vitro. Our molecular dynamics simulations of the fimbrial tip structure show that FimH is shaped like a hook that is normally rigid but opens under force, causing structural changes that lead to firm anchoring of the bacteria on the surface. In contrast, the more distal adaptor proteins of the fimbrial tip create a flexible connection of FimH to the rigid fimbria, enhancing the ability of the adhesin to move into position and form bonds with mannose on the surface. We suggest that the entire tip complex forms a hook-chain, ideal for rapid and stable anchoring in flow.

## Introduction

Most adhesive biological processes are exposed to mechanical stress resulting from fluid flow-induced shear. Thus, the molecular structures that mediate adhesive interactions are adapted to function in mechanically dynamic conditions. In the case of gram-negative bacterial cells, the interaction with the host tissue is known to be mediated by adhesive proteins (adhesins) that are, in many cases, positioned at the tip of multimeric hair-like appendages called fimbriae (or pili) and bind to receptor molecules on the target cells or tissues [1],[2]. The 30 kDa FimH protein is the most common, mannose-specific adhesin of *Escherichia coli* located on the tip of type 1 fimbriae [3],[4].

Bacterial adhesion mediated by type 1 fimbriae is enhanced by shear stress [5],[6], and single molecule force spectroscopy experiments have shown that a tensile force extends the lifetime of the bond between FimH and the mannose receptor [7]. The force-enhanced, so-called catch bond mechanism of FimH binding involves allosteric activation of the mannose-binding lectin domain (Ld), which switches from a low- to a high-affinity conformation upon separation from the anchoring pilin domain (Pd).

**Figure 1:**
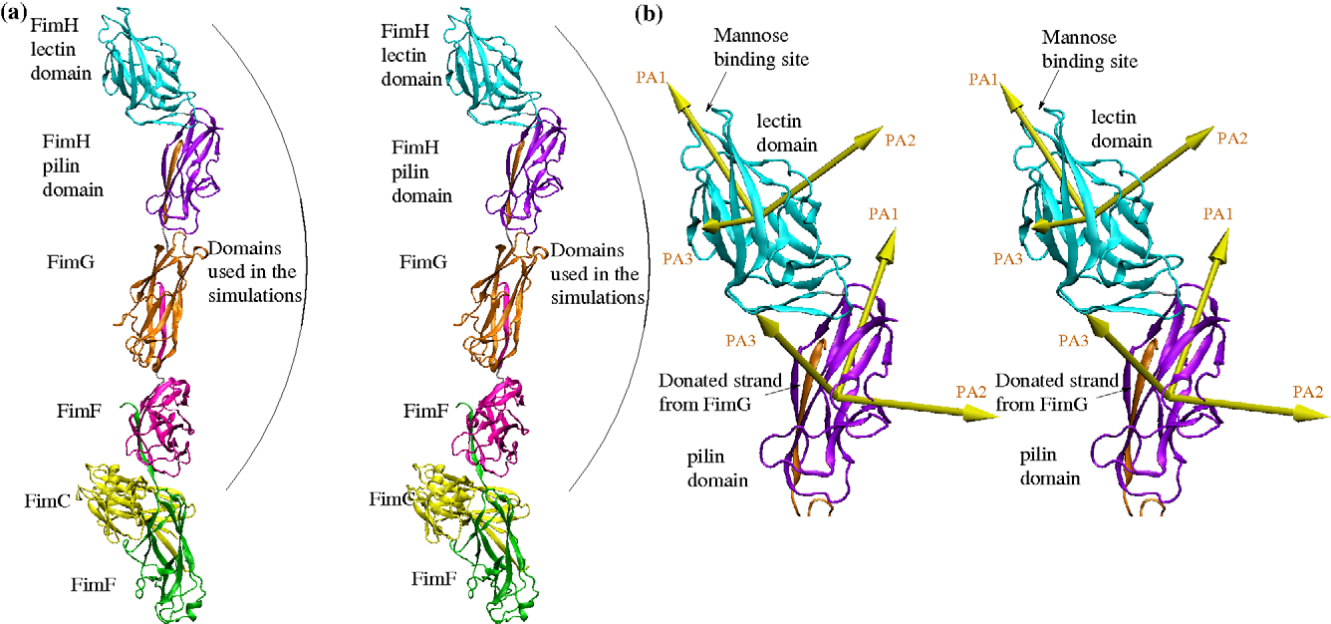
Crystallographic structures. (a) Entire fimbrial tip (stereoview). Subunits are distinguished using different colors. A subunit consists of a polypeptide chain, whereas a domain is defined as the globular part of a subunit and (with the exception of the FimH lectin domain) a donated strand of the neighboring subunit (see “Materials and Methods” for the exact definition of start and end residues of domains). The FimH subunit consists of two domains, which are also distinguished with different colors: cyan for the lectin and purple for the pilin domain, respectively, except for the donated strand of FimG, which is colored orange as the subunit it belongs to. The two FimF subunits are colored magenta and green, respectively. The chaperone protein FimC is in yellow. Residues that are located between domains define linker chains and their backbone is colored in silver. The arrows in yellow represent the principal axes (PA1, PA2, and PA3) of each domain (see “Materials and Methods” for the determination of principal axes). They are used to calculate hinge and twist angles between adjacent domains (Figures S3, S4, S5d,e). (b) FimH subunit with donated FimG strand (stereoview). The principal axes of the FimH subunit are labeled in brown.

The type 1 fimbria consists of a 1–2 µm long fimbrial rod, which is built by thousands of copies of the non-adhesive major subunit FimA, and the fimbrial tip, which comprises three minor subunits, i.e., FimF, FimG, and the FimH adhesion [2],[3],[8]. Several crystallographic and nuclear magnetic resonance studies have investigated the structure of the monomeric or dimerized minor subunits [9]–[14]. Most of these studies were performed with FimH, either with isolated Ld [14] or with the entire protein in complex with the chaperone FimC wedged between Pd and Ld [9],[11]. Recently, the X-ray structure of a native form of FimH was obtained, where the FimH adhesin is incorporated into the fimbrial tip complex comprised also of one FimG and two FimF subunits, with FimC wedged between the two FimF copies (Figure 1a) [8]. Importantly, when comparing the tertiary structure of Ld in the tip-incorporated FimH with that in the isolated Ld or FimH/FimC complex, some remarkable conformational differences were observed [8]. In the tip complex, Pd is docked onto Ld causing compression of the β sandwich fold of Ld by twisting two β sheets relatively to one another. The mannose-binding pocket is located on the opposite side of the binding domain relative to the Pd/Ld inter-domain region (Figure 1b), but the twisting in the inter-domain region leads to opening of the mannose pocket because the rigidity of the β sheet transmits structural perturbations over long distances [8]. In contrast, when Pd is separated, Ld assumes an elongated, less twisted conformation with a tight conformation of the mannose-binding pocket that has a more than 200-fold higher affinity to mannose than the low-affinity, compressed conformation of Ld [5]. It has been suggested that FimH Ld functions like a molecular finger-trap that switches from a low- to a high-affinity conformation upon separation from Pd, which is caused by tensile mechanical force originated by shear stress [8]. Such force-induced activation of the FimH adhesin is the basis of the shear-enhanced catch bond mechanism of the type 1 fimbriae-mediated bacterial adhesion under flow [5].

While the tertiary structure of Ld has been the primary focus of a recent study [8], the dynamic mechanical properties of the entire tip complex have not been studied and it is not clear how the quaternary structure of the tip is adapted to function under shear conditions. Using a combination of flow chamber experiments, single molecule force spectroscopy, and molecular dynamics (MD) simulations, we investigated here how the structural properties of all fimbrial tip components are optimized to facilitate the initial interaction of FimH with the surface receptor, its switch to the activated form, and then its sustained binding under dynamic flow conditions. In summary, the fimbrial tip acts as a mechanical force sensor.

## Results

### Functional Properties of the Fimbrial Tip

The protein FimH is generally highly conserved in different *E. coli* strains, though naturally occurring point mutations are known to make binding less dependent on shear by increasing the adhesion under low or no flow conditions [15]. While such mutant variants are found among uropathogenic strains, the bulk of FimH variants among all *E. coli* pathotypes, including uropathogenic ones, clearly demonstrate shear-dependent binding. In particular, the FimH variant crystallized in the fimbrial tip represents the most common protein variant found in *E. coli* causing extra-intestinal infections and belonging to the so-called B2 clonal group.

**Figure 2:**
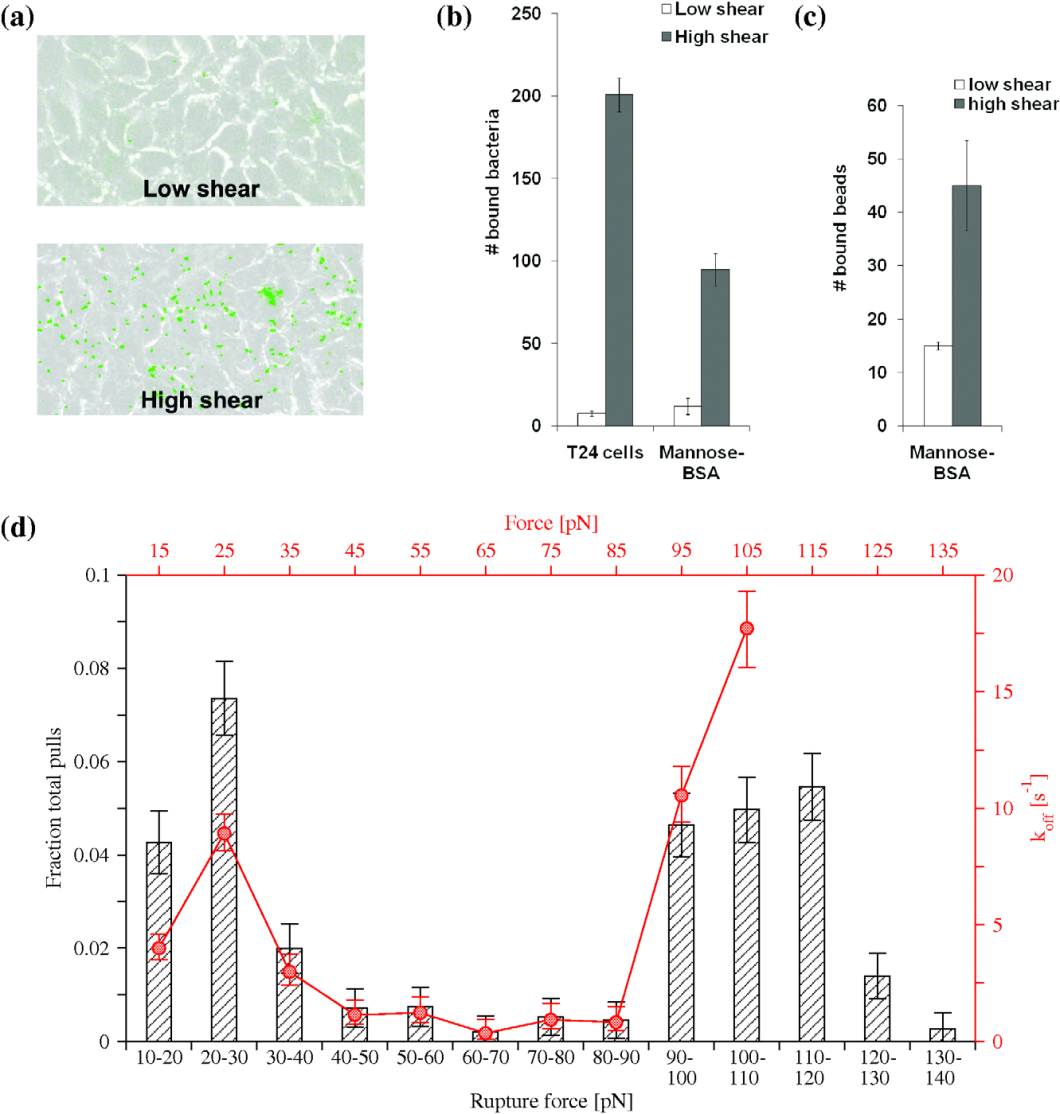
Shear-enhanced adhesion and catch bond behavior. (a) Binding of *E. coli* to uroepithelial cells at low (0.01 Pa) and high (0.1 Pa) shear stress in a flow chamber. (b) Level of *E. coli* binding under low (0.01 Pa) and high (0.1 Pa) shear stress to uroepithelial cells and mannose-BSA coated surface. (c) Binding of fimbrial tip-coated beads to mannose-BSA coated surface. (d) Binding of fimbrial tips to mannose-BSA in single molecule force spectroscopy experiments. The histograms in black (ordinate on the left, abscissa at the bottom) show the fraction of total pulls rupturing within a bin of a force range. The red line (ordinate on the right, abscissa at the top) displays the calculated unbinding rate (k_off) as a function of the force.

The shear-dependent properties of the tip-incorporated FimH have been demonstrated previously using either yeast mannan, mannose coupled to bovine-serum-albumin (BSA), or guinea pig red blood cells [5],[7],[16],[17], all of which are surrogate receptors for the type 1 fimbriae [15]. We tested whether the type 1 fimbriae mediate shear-dependent adhesion to a natural target like bladder epithelial cells. Bacteria expressing type 1 fimbriae, with FimH, FimG, and FimF structurally identical to the ones in the crystallized tip complex, were used in parallel plate flow chamber experiments over the monolayer of bladder cell line T24. Bacterial adhesion to the cells increased more than 20-fold when shear was switched from 0.01 Pa to 0.1 Pa (Figure 2a,b). The pattern of *E. coli* adhesion to uroepthelial cells under different shears was similar to the bacterial binding to mannose-BSA coated on a surface (Figure 2b), indicating the monomannose specific mechanism of the shear-dependent *E. coli* adhesion to the bladder cells. Moreover, purified fimbrial tips that were used for the X-ray studies, when coupled to plastic beads, also mediated shear-enhanced binding to a mannose-BSA coated surface (Figure 2c).

We then used single molecule force spectroscopy to establish how the bond between the fimbrial tips and mannose responds to various amounts of mechanical force. Previous single molecule force spectroscopy experiments with fimbrial tips never resulted in a measure of the dissociation rate as a function of force. This is because the bond lifetime was too long (many minutes) to be measured one molecule at a time in constant force experiments [8] and because the alternative approach, a constant loading rate, had not been analyzed in a way that calculated the dissociation rate and was also performed on different variants of FimH [7]. Here, a new method was used to analyze constant loading rate experiments to estimate dissociation rates at various levels of force [18]. This method also shows the catch bond behavior in a more direct, intuitive fashion than either previous method. The force was increased at a constant loading rate on bonds between fimbrial tips and mannose-BSA to obtain histograms of rupture forces (Figure 2d). The instantaneous dissociation (off-) rate was then estimated from the number of bonds that break relative to the number remaining for each bin. This method provides a measurement of the force dependence of the effective off-rate using a single constant loading rate. A loading rate of 300 pN/s was previously shown to provide an even distribution between the low and high force peaks in the histogram [7], and thus was used here to provide adequate statistics in both regions. This force-dependent effective dissociation rate (red line in Figure 2d) shows that the off-rate decreases upon the force increasing between 30 and 80 pN (before beginning to increase above 90 pN). The existence of a regime in which increased force decreases the dissociation rate is the modern definition of catch bonds [19],[20]. Thus, taken together, these results indicate that the fimbrial tip complex used for the X-ray analysis [8] exhibits shear-enhanced binding to uroepithelial cells under flow conditions and catch-bond behavior under tensile mechanical force.

### Quaternary Structure of the Fimbrial Tip

To predict how the components of the tip complex might behave under dynamic force conditions, we first evaluated the overall quaternary structure of the tip based on the X-ray data. In the fimbrial tip, Pd is connected to FimG, FimG to FimF, and FimF to another copy of FimF via a donor-strand complementation mechanism [21], where a missing β strand in each β sandwich shaped subunit is complemented by an N-terminal strand of the following subunit. The linkage via the complementing strand mechanism is among the strongest of non-covalent bonds and, thus, to ease the presentation we will refer here to the FimG and FimF subunits (with the complementing strands) as domains, similarly to the Ld and Pd in FimH that are covalently linked to each other via a linker chain, and Pd includes also the FimG donor strand (see also Figure 1a and “Materials and Methods”).

A striking feature of the quaternary conformation of the fimbrial tip crystal structure is the end-to-end position of all domains relative to one another that results in an extended structure of the tip complex, with a total length of approximately 230 Å (Figure 1a). However, the extent of quaternary interaction in the various inter-domain interfaces is drastically different.

**Figure 3:**
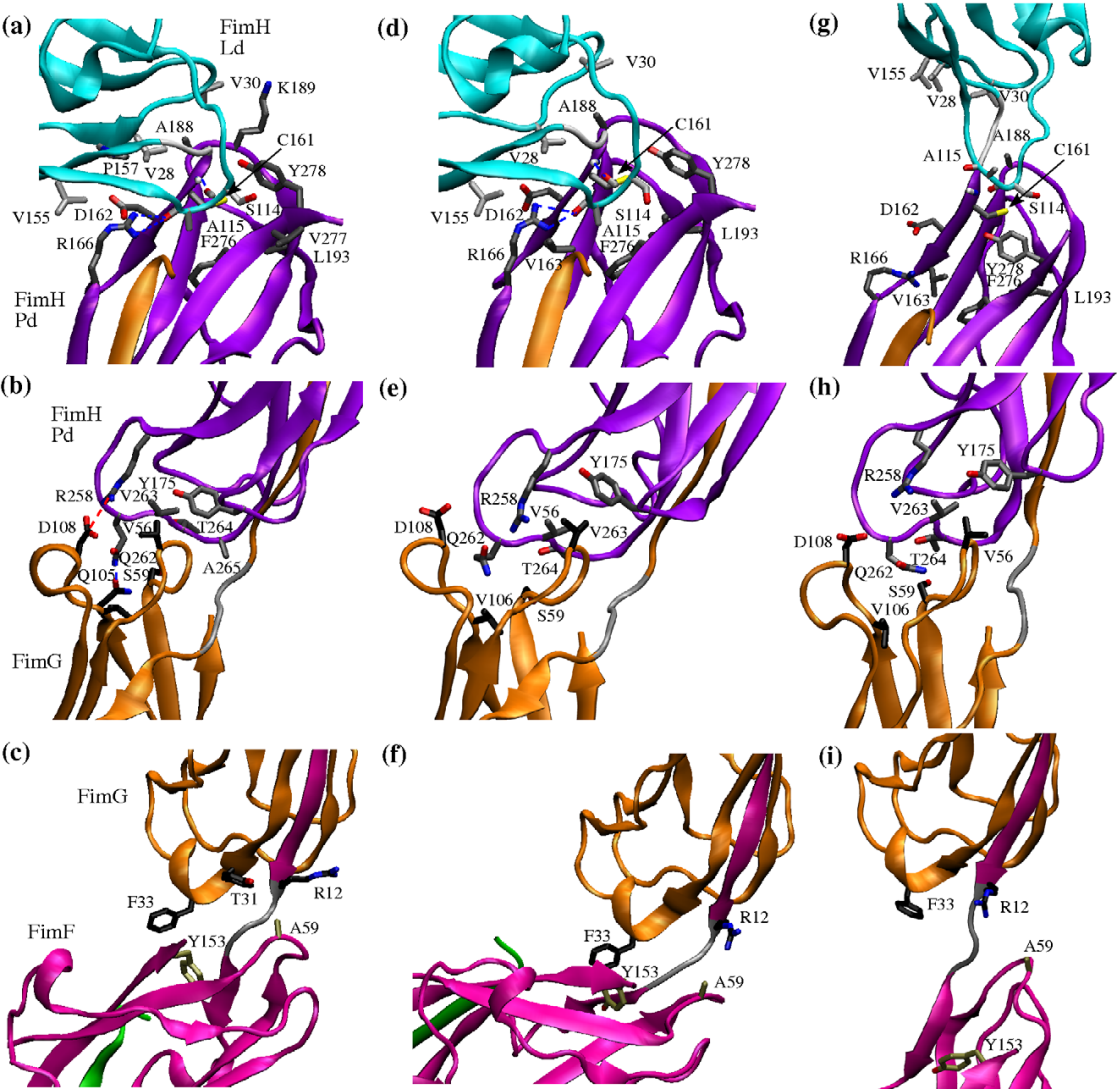
Inter-domain side chain and electrostatic contacts in MD simulations. (a, b, and c) Inter-domain interfaces in the X-ray structure. (d, e, and f) Conformation after 14 ns in a 300 K simulation with two neighboring domains (the conformation in (d) is taken from the 40 ns run). (g, h, and i) Conformation after 10 ns in a pulling simulation. Side chains involved in contacts or salt bridges or hydrogen bonds are displayed in the stick and ball representation (see “Materials and Methods” for the definition of side chain and electrostatic contacts). Backbone atoms forming hydrogen bonds are also represented. In the central and right column, those contacts are displayed that are observed to be persistent in the 300 K simulations (i.e., they occur in at least 66% of the simulation frames, see “Materials and Methods”). Side chains involved in persistent contacts are labeled only in the central column. The carbon atoms of the displayed side chains are colored differently depending upon which domain they belong to: light grey for the lectin domain, dark grey for the pilin domain, black for FimG, and tan for FimF. The domains are colored as in Figure 1. The figure was prepared with VMD [60].

While the wedged FimC chaperone made it difficult to evaluate the FimF-FimF interface, the buried surface between FimF and FimG (501 Å^2^) was smaller than between FimG and Pd (760 Å^2^) and even smaller than the interface buried between Ld and Pd (1,040 Å^2^; see “Materials and Methods” for a description of how the surface buried between domains was calculated). There were only three side chain interactions and no hydrogen bonds or salt bridges between the FimF and FimG domains (Figure 3c), while between FimG and Pd there were six side chain contacts, one hydrogen bond, and one salt bridge (Figure 3b). The most extensive interactions were between Pd and Ld, with 14 side chain contacts and three hydrogen bonds, with one of them involving backbone atoms, i.e., Cys161 NH … O Ser114, and two involving the side chain of Arg166 and the carboxyl oxygen of Ala115 (Figure 3a and Table 1).

**Table 1:** Number of inter-domain contacts.

Interface	X-Ray^a^	300 K Native^b^	300 K Average^c^	Pulling^d^
**Side chain interactions**				
Ld-Pd	14	9 (9)	8.54±0.73	0.98±0.14
Pd-FimG	6	7 (6)	6.19±0.8	5.62±0.88
FimG-FimF	3	2 (2)	1.44±0.56	0.1±0.3
**Hydrogen bonds**				
Ld-Pd	3	3 (3)	2.6±0.56	0.58±0.5
Pd-FimG	1	0	0	0
FimG-FimF	0	0	0	0
**Salt bridges**				
Ld-Pd	0	0	0	0
Pd-FimG	1	0	0	0
FimG-FimF	0	0	0	0

**Figure 4:**
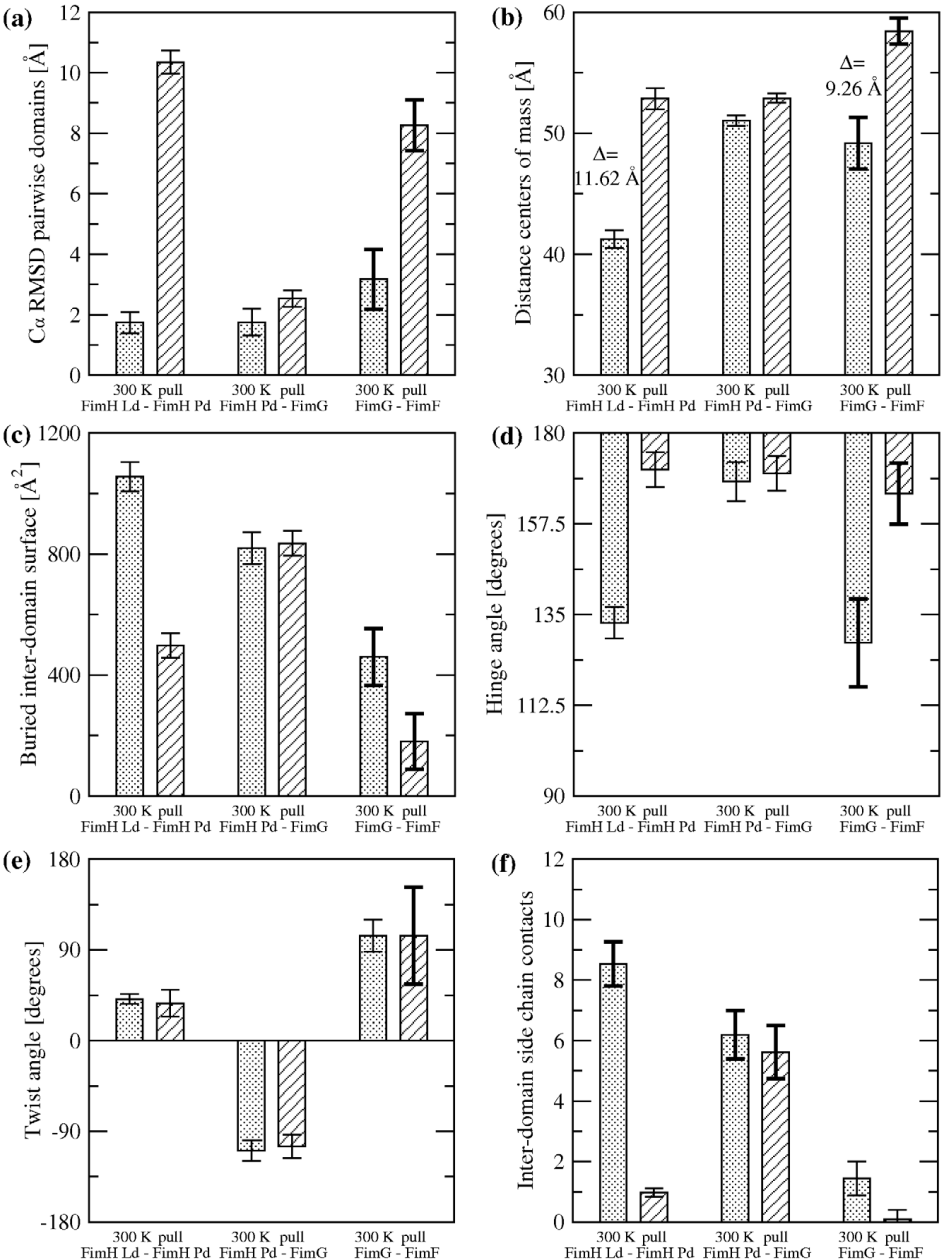
Quantitative data from MD simulations. Bars filled with dots indicate averages of quantities measured during the 300 K simulations, while bars with angular hatching indicate averages from the last 2 ns of pulling runs. Error bars show standard deviations (SD). To highlight quantities with large SD, the error bar is thicker if the SD is larger than the average of all SDs of a given quantity. (a) Cα RMSD of pairwise adjacent domains. (b) Distance between the centers of mass of two adjacent domains. (c) Surface area buried between two adjacent domains. (d) Hinge and (e) twist angle between two adjacent domains, respectively. (f) Number of native side-chain contacts between two adjacent domains.

See “Materials and Methods” for a definition of side chain contacts, hydrogen bonds, and salt bridges.

a Contacts observed in the crystallographic structure.

b Contacts present in at least 66% of the simulation frames of the 300 K runs (referred to here as native contacts), excluding the first 10 ns, which are considered equilibration (the number in parentheses refers to the subset of native contacts observed also in the X-ray structure).

c Average and standard deviation of the number of native contacts during the 300 K runs, excluding the first 10 ns (see also Figure 4f).

d Average and standard deviation of the number of native contacts during the last 2 ns of the pulling simulations (see also Figure 4f).

Besides the largest buried inter-domain interface, there was another notable feature of the Ld-Pd quaternary conformation, namely it had a “hooked” conformation. The domains Ld and Pd were hinged at an angle of 128° (Figure 1b). In contrast, the angle between the other extensively interacting domains, Pd and FimG, was almost completely open at an angle of ca. 169° (Figure 1a and Figure S1a). In summary, the proximal (i.e., closer to the fimbrial rod) part of the tip has significantly fewer interdomain contacts relatively to the distal portion of the tip, which is also characterized by a distinctly hooked conformation of Ld and Pd.

### Tip Flexibility in the Absence of Tensile Force

In order to test the flexibility of the different interdomain interfaces, MD simulations were performed with three pairs of neighboring fimbrial tip domains: Ld-Pd, Pd-FimG, and FimG-FimF (Table 2). The quaternary conformation of the complexes Ld-Pd and Pd-FimG was mostly stable in the simulations, with the Cα root mean square deviation (RMSD) from the initial conformation remaining below 3.5 Å for both complexes (Figure 4a and Figure S2a). In contrast, the Cα RMSD for the FimG-FimF complex exceeded 5.5 Å in the course of the simulation (Figure 4a and Figure S2a), indicating that the quaternary structure of these domains is relatively flexible. The observed flexibility is mainly due to rigid body movements of the domains relative to each other, since the Cα RMSD for all domains individually was mostly below 2 Å (Figure S2b). Similarly, the distance between centers of mass of neighboring domains and the amount of buried surface area in the inter-domain interface remained constant for the Ld-Pd and Pd-FimG complexes but fluctuated notably in the FimG-FimF complex (Figure 4b,c and Figure S2c,d).

**Table 2:** Simulation systems.

Simulated Domains^a^	Force^b^	Duration	Description
FimH Ld+FimH Pd		40 ns+27 ns+25 ns	Three 300 K runs^c^
FimH Ld+FimH Pd		27 ns	300 K run
FimH Ld+FimH Pd		25 ns	300 K run
FimH Pd+FimG		28 ns	300 K run
FimG+FimF		28 ns	300 K run
FimH Ld to FimF	200 pN	10 ns	Pull_1^d^
FimH Ld to FimF	200 pN	10 ns	Pull_2^d^
FimH Ld to FimF	200 pN	10 ns	Pull_3^d^
FimH Ld+FimH Pd	200 pN	10 ns	Pull_FimH_1
FimH Ld+FimH Pd	200 pN	10 ns	Pull_FimH_2
FimH Ld+FimH Pd	200 pN	10 ns	Pull_FimH_3

a Domain structures used to start the simulations; includes also the linker chain covalently connecting the domains.

b The simulations where no force was applied were used to study the flexibility of the domains at room temperature and determine persistent inter-domain contacts.

c All three simulations with the FimH domains were started from the X-ray structure, but different velocities were assigned to the atoms at the beginning of each run.

d The pulling runs with the fimbrial tip were started from the conformation obtained after equilibrating the structure at 300 K during 3 ns.

In order to further describe the movement of the domains relative to each other, the angles between the principal axes of the domains were monitored. The angle between the first principal axes (PA1 in Figure 1b) subtracted from 180° is called the “hinge angle,” whereas the angle between the second principal axes (PA2 in Figure 1b) is labeled “twist angle.” (The angle between the third principal axes, PA3 in Figure 1b, was observed to essentially correlate with the twist angle and thus was not monitored.) The FimH domains remained in the hooked conformation throughout all runs and the hinge and twist angles measured 133°±4° and 41°±5°, respectively. The Pd-FimG complex also retained its conformation, with a similar amount of variation in the hinge angle (168°±5°) but a slightly more pronounced variability in the twist angle (−109°±10°; the negative values are due to the location of the donor strand complementation grooves in Pd and FimG on opposite sides). The highest variability was between FimG and FimF, where the hinge angle measured −52°±11° and the twist angle was 104° and fluctuated with a standard deviation of 16° (Figure 4d,e and Figure S2e,f), supporting the hypothesis that the FimG-FimF interface is very flexible and explores a relatively large space compared to the other domains.

The differences in flexibility between the three complexes, with Ld-Pd being the most rigid and FimG-FimF the most flexible, are consistent with the number of native inter-domain interactions (Figure 3d–f, Table 1, and Figure S2g). The interface between Ld and Pd (Figure 3d) presents the largest number of native side chain contacts and hydrogen bonds (Table 1), the latter being buried throughout the MD simulations.

### Conformational Changes under Tensile Force

In order to investigate the conformational changes induced by a tensile force onto the fimbrial tip, pulling simulations were performed with the complex containing Ld, Pd, FimG, and the first copy of FimF (Figure 1). A constant force of 200 pN was applied for 10 ns in three separate simulations between mannose-binding site residues on the apex of Ld and the C-terminus of the donor strand connecting the two FimF subunits (Table 2).

Most conformational changes were observed in the first 2 ns during the pulling runs (Figures S3, S4, S5) with the tip extending in total 37 Å (22% of the native structure). The most stable interdomain interface during the pull was between Pd and FimG, which remained almost unchanged, keeping a similar geometry as the native state (Figure 4 and Figures S3, S4, S5). In contrast, the quaternary structure of FimG-FimF underwent substantial conformational changes, with an increase of the Cα RMSD from the native conformation (Figure 4a), an increase in the distance between the centers of mass of the domains (Figure 4b), and a virtual elimination of the buried surface area (Figure 4c) and native side chain contacts (Figure 4f and Figures S3, S4, S5). Also, after the straightening under tensile force, the quaternary structure of the FimG-FimF complex was observed to be much more flexible than in the absence of force, as indicated by the large fluctuations in the twist angle (Figure 4e).

**Figure 5:**
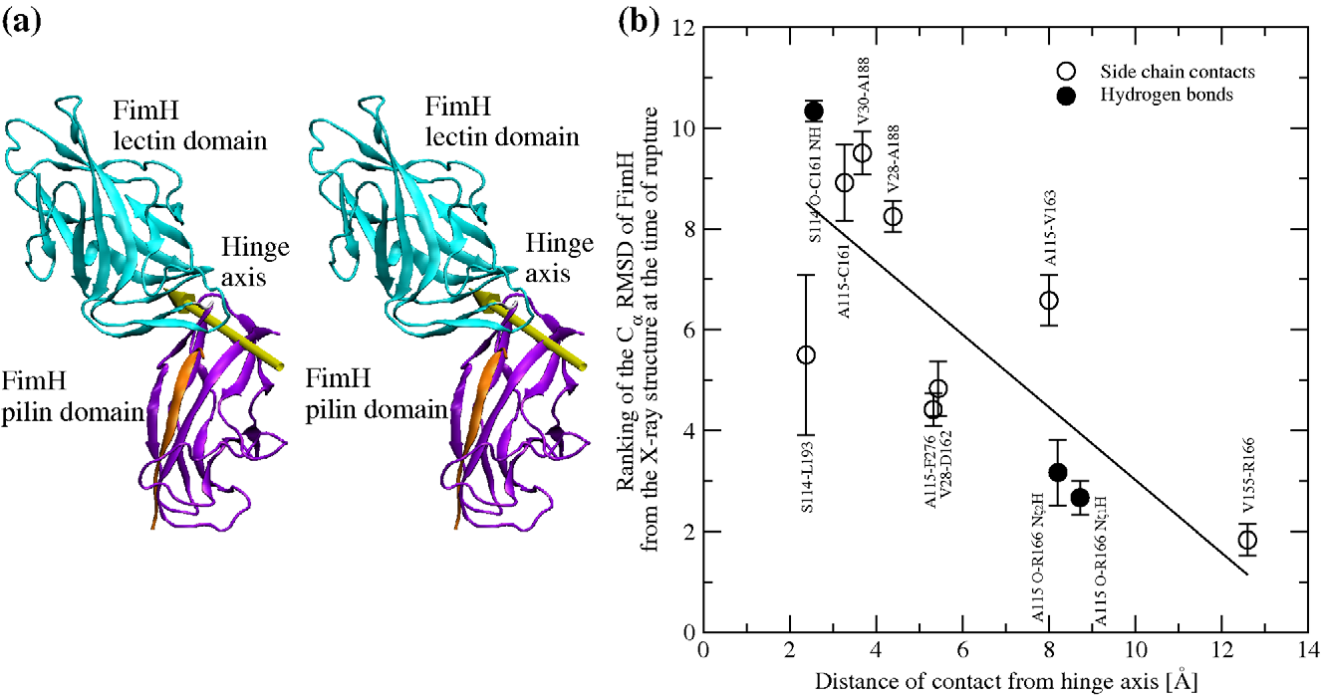
Unhinging pathway of FimH under tensile force in MD simulations. (a) Location of the hinge axis (stereoview) determined with the program DynDom [61] by comparing the conformation of FimH at the end of the run pull 1 with its native conformation. (b) Sequence of rupture events of contacts between Ld and Pd versus distance from the hinge axis. A contact was defined as broken at the first time point when it ruptured and was not seen to reform within 300 ps. The Cα RMSD was calculated at the time of rupture (time averaged over 200 ps, Figures S3a, S4, S5a) in all six pulling runs and ranked according to a fractional ranking algorithm (where equal values receive the same raking as their respective ordinal rankings). The average and standard error of the mean of the ranked RMSD values are plotted against the distance of the respective contact from the hinge axis. The Cα RMSD from the native structure is a better measure of progress than the time of rupture itself, because rupture events are observed to occur in approximately the same order in every simulation but the time point when they occur varies across the simulations. The distance of a contact from the hinge axis is calculated as the distance of the geometric center of the involved side chains from the axis (in the case of hydrogen bonds, the geometric center of the D–H … A atoms is used). Significant inverse linear correlation (Pearson's ρ = 0.78; *p* value = 0.004) shows that the ranking number decreases with increasing distance, suggesting that the larger the distance from the hinge axis, the earlier the rupture of a contact.

However, the most drastic changes occurred in the Ld-Pd structure, where the inter-domain hook straightened to an almost flat angle (Figure 4d and Figure S1e), with a large increase in the distance between the centers of mass of the domains, a significant decrease in buried surface area, and elimination of most native contacts (Figure 4 and Figures S3, S4, S5, S6, S7, S8, S9). In order to improve the statistical sampling of the events occurring during separation of the lectin from the pilin domain, three additional pulling simulations were performed with just the FimH protein. In both sets of pulling runs the rupture of inter-domain contacts between Ld and Pd happened in a sequential manner (Figure S10). Importantly, the combined results analysis indicates that the order of the contact breakage was inversely correlated with the contact's distance from the hinge axis, i.e., bonds further away from the hinge ruptured earlier in the simulations than contacts located closer to it (Figure 5). The Pearson's linear correlation coefficient was 0.78 with a *p*-value<0.01. This suggests that Pd and Ld unzip under tensile force, where only one or a few inter-domain contacts break at a time instead of most or all of the contacts breaking simultaneously. The fact that the sequence of breaking events is statistically correlated with the distance of the contacts from the hinge axis is indicative that the hooked shape of FimH allows a sequential unzipping of the stabilizing contacts as the hinge opens.

In conclusion, these simulations demonstrate that mechanical force can relatively easy and in an unzipping manner open the hinge angle between the two domains and eliminate the native interdomain contacts. These native interdomain contacts were implicated previously in allosterically maintaining the low-affinity conformation of Ld, so that loss of these contacts should lead to activation [8]. Allosteric conformational changes are not expected to be observed in molecular dynamics simulations due to the short time scale of the simulations (10 ns) relative to that of typical allosteric changes (microsecond to millisecond), so this cannot be directly confirmed in the simulations. Nevertheless, these results strongly suggest that FimH would allosterically switch its conformation from low to high affinity once the hinge opens.

### Mechanics of the Fimbrial Hook

Several independent measurements showed previously that most FimH bonds dissociated within a second if little or no force was applied across the complex, but most bonds were long-lived under a sufficient tensile force load [8]. Thus, mannose unbinding from the low-affinity state is the dominant event at no and low force, but stable binding to the high-affinity (activated) state appears to dominate at high force. While the pulling simulations show that tensile force *can* open the hinge angle enough to lead to FimH activation, it remains to be addressed whether opening of the hinge angle will occur before mannose is pulled out from the pocket.

**Figure 6:**
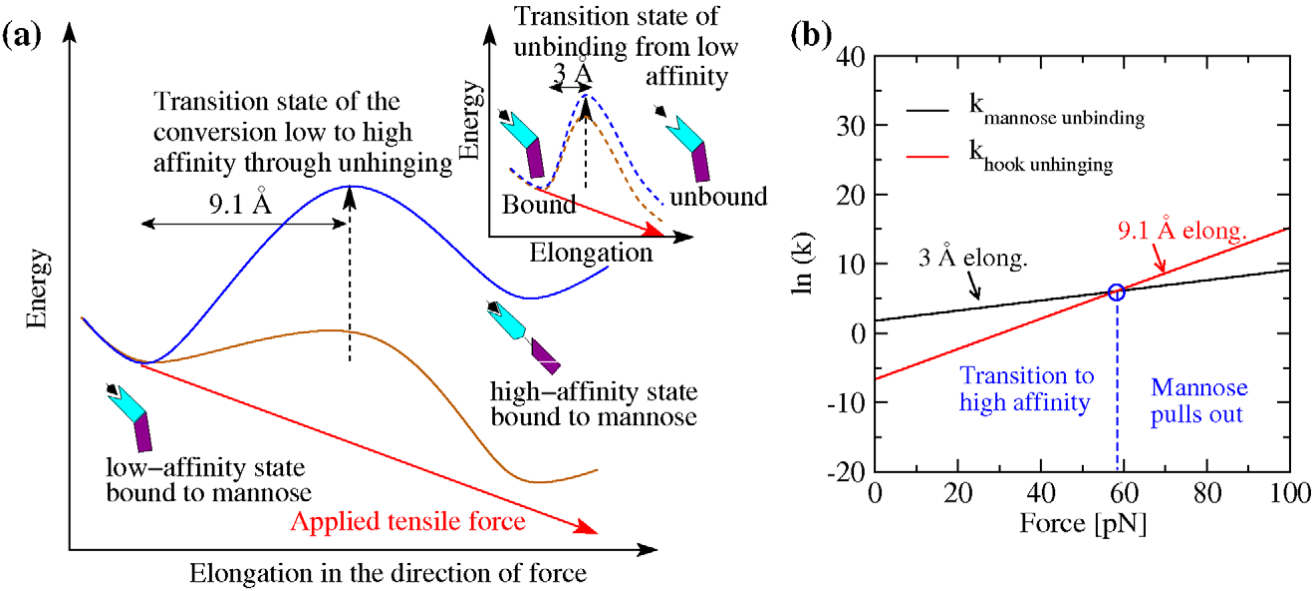
Effect of force on dissociation kinetics versus unhinging and activation kinetics. (a) Schematic representation of the energy landscape of FimH bound to mannose in the absence of force (blue) and in the presence of an external tensile force (brown). The energy barrier for the unhinging conversion from the low- to the high-affinity state is represented as solid lines. The barrier of the mannose unbinding process from the low-affinity state is shown in the insert as dashed lines. The energy added to the system as a result of the applied tensile force is indicated by a red line. The energy landscape in the presence of force is a result of the superposition of the energy landscape in the absence of force and the product of force times elongation. The elongation between the native and transition states is indicated. (b) Representation of the slopes for the rate constants for unhinging and unbinding. Since the elongation until conversion to the high-affinity state is not known, the slopes are calculated for the lower limit, 8.5 Å, and the upper limit, 18 Å, of the range. The intersection between the slopes for the unhinging rate and the slope for the mannose unbinding rate are indicated by blue circles. Where the slopes intersect the rate of unbinding and the rate of unhinging is equal.

According to kinetic rate theory [22], the rate at which a physical event occurs—in our case, dissociation of mannose or, alternatively, opening of the hinge angle between the FimH domains—is exponentially related to the size of the energy barrier ΔE of the reaction transition state relative to the original equilibrium state of the system: k^0^ = A exp [−ΔE/k_B_T], where A is the Arhenius prefactor and k_B_T is thermal energy. However, mechanical force can speed up a reaction by pulling the protein into the transition state if it is elongated relative to the native state. We define here Δx as the increase in length between the transition state and the native state, thermally averaged and projected onto the direction of force. Then, a constant force field contributes an amount of energy F Δx, to help overcome the transition state energy barrier, effectively decreasing its size as illustrated in Figure 6a. This exponentially increases the reaction rate: k(F) = k^0^ exp [F Δx/k_B_T] [23]. Thus, the larger the elongation distance, the greater will be the effect of the same force onto the reaction rate. To determine how force affects FimH, we thus need to know the elongation distances for the two transitions in question: mannose-unbinding versus hinge opening and activation.

Transition state elongation distances can be estimated from MD simulations [24], which provide structural details explaining force dependence in force spectroscopy data [25]. To measure elongation distances from the pulling trajectories, we estimate the location of the transition state and then measure the total elongation necessary to reach this state from the native state. The transition state for mannose to be pulled out of FimH (i.e., breaking of the bond) consists of the mannose molecule being shifted from a horizontal into a perpendicular position within the binding pocket, elongating the Ld-mannose complex approximately 3 Å [26]. The minimal length change for the alternative transition state, where the domains unhinge and Ld activates, was estimated from the pulling simulations above. Screening of the trajectories revealed that the rupture of inter-domain contacts involving the side chain of Arg166 (a side chain contact with Val155 and the hydrogen bonds with the carbonyl oxygen of Ala115) was the first rupture event observed in all pulling simulations (Figures S6, S7, S8, S9). Since the protein elongated essentially monotonically, any later events would involve even greater elongation distances. For this reason, the Arg166 rupture event provides a lower limit for the location of the dominant transition state. The importance of this event is further strengthened by the fact that mutations of Arg166 cause strong activation of FimH [24]. The elongation distance associated with this event was calculated to be 9.1±1.2 Å as measured between the N-terminus of FimH and the C-terminus of FimG (Table S1 and “Materials and Methods” for details on the calculation of this distance).

It follows that the activation pathway involves a longer elongation than does the mannose unbinding pathway, since the lower limit of 9.1±1.2 Å of the former is greater than 3 Å of the latter. Thus, increased force will favor activation. To provide an intuitive idea of how important this difference in length is, Figure 6b depicts the effect of force, given the transition state elongation distances described above, on the rate constants for the two transitions. For this estimation, the rates in the absence of force were taken from previous experimental results to be 6 s^−1^ for mannose unbinding [16] but only 0.00125 s^−1^ for opening of the hinge angle [27]. At an elongation distance of 9.1 Å, the rate of hinge opening surpasses the rate of unbinding at a critical force of 58 pN (Figure 6b). If the rate limiting step occurs after the Arg166 separation, the larger elongation distance will mean a steeper slope in Figure 6b and a lower critical force. Thus, unbinding dominates below a critical force of 58 pN, while hinge opening and activation dominates above the critical force.

The critical force determined by the calculations presented here is consistent with single molecule force experiments (Figure 2d) where the bond between the fimbrial tip and mannose is observed to become high affinity at similar force magnitudes. This supports the model that the hook opening leads to the switch from the low- to the high-affinity state, significantly slowing the mannose unbinding rate [8]. The hook shape of FimH thus ensures that there is a much larger distance, and thus a greater responsiveness to force, for hinge opening relative to mannose unbinding. This physical property allows a tensile force to activate FimH rather than pulling mannose out of the pocket.

## Discussion

A long-standing assumption about the microbial adhesive organellae is that their quaternary conformation should be flexible to not impede the ability to explore a target surface in order to quickly find the corresponding receptor molecule and thereby maximize the effectiveness of the adhesins' function. For so-called class 1 fimbriae, where the adhesin is located on the organella's tip (like in the type 1 fimbriae or di-galactose-specific P fimbriae), the quaternary structure of the multi-protein fimbrial tip complex was also proposed to be highly flexible to optimize the binding rate [28]. Indeed, high levels of mobility have been observed previously in NMR studies of FimG-FimF and FimF-FimF dimers in the type 1 fimbrial tip [10]. Furthermore, in the original X-ray structure of FimH that was obtained in complex with FimC [9], the Ld and Pd were separated from one another and, because it was then believed that the separated domains conformation is native, this was also attributed to the need of high mobility of the mannose-binding Ld. Mobility is likely to be important in the fimbrial tips because the main shaft of the type 1 fimbriae is rigid [3] and thus would diffuse slowly within a very restricted exploration space between the bacteria and the target cell surface. However, considering that type 1 fimbriae and other bacterial adhesins are able to mediate shear-enhanced adhesion via the formation of catch bonds [7],[29], the adhesive organellas not only need to be adapted to ensure high mobility of the adhesin, they must also have an effective mechanism to permit the adhesins to be activated by tensile force. As shown in this article, this results in more complex than previously thought mechanical properties of the quaternary structure of the adhesion apparatus.

The primary structure of the 300 amino acid-long FimH is 99% conserved across *E. coli* strains. The vast majority of the naturally occurring FimH variants from fecal and pathogenic *E. coli* mediates well-manifested shear-dependent adhesion. Though FimH in uropathogenic *E. coli*, especially in the strains causing infection of kidneys, is under positive selection to acquire mutations that increase binding at static or low-shear conditions, most of the FimH variants in uropathogenic strains still manifest shear-dependent phenotype to at least some extent [30]–[32]. For example, the FimH variant crystallized in previous studies [9],[11], which was obtained from a model uropathogenic strain J96 and is a common natural variant, exhibits a distinct shear-dependent binding [29],[32]. The FimH protein crystallized in the fimbrial tip complex [8] and analyzed here differs from the former variant in three amino acids (A27V, S70N, N78S) but is an even more common (and, actually, evolutionary primary) variant among *E. coli* that causes extra-intestinal infections in humans, including urinary tract infections [15]. FimH variants with completely shear-independent properties are relatively rare and appear to be selected out very fast outside the urinary tract [31].

Using *E. coli* expressing the tip-associated FimH variant, we showed here that bacterial adhesion to the bladder uroepithelial cells increases 20-fold when shear increases from 0.01 to 0.1 Pa. Physiological shear stress along the bladder surface is difficult to evaluate and can result from the flow dynamics of urine as well as from the bladder stretching/contraction. The flow-derived level of wall shear stress equals 4Vμ/πR^3^, where V is volumetric flow rate, μ is fluid viscosity, and R is the radius of the tube. In the human urinary tract, one can estimate that shear stress is quite low in the ureter (0.001 Pa, based on a 3 mm diameter and 0.01 ml/s flow) but can reach 0.3 to 0.5 Pa in the urethra in the course of urination (based on the average urethra diameter of 5–6 mm and urine flow of 20–30 ml/s). Thus, though the shear stress along the bladder surface is expected to vary dramatically depending on the specific compartment or contraction state of the bladder, the shear stress levels tested here are likely to be within the physiological range. These levels are also within the range observed in other compartments. For example, shear along vascular endothelial cells ranges from 0.1 to 0.2 Pa on the venous side and 1 to 2 Pa (up to 5 Pa) on the arterial side of the circulation [6],[7]. Shear stress generated at the tooth surface by salivary flow is approximately 0.08 Pa [8]. Shear stress in the intestines is estimated at 1 to 2 Pa (or higher with viscous lumen) [33] due to peristalsis [34].

The force-enhanced, catch bond fimbrial properties are ultimately based on the ability of the binding domain of FimH, i.e., Ld, to assume two conformational states, a twisted compressed form with a low affinity and an elongated form with a high affinity toward mannose. In turn, these states are intimately linked to whether Ld is docked to or separated from the anchoring domain of FimH, i.e., Pd. Thus, the effectiveness of FimH in mediating bacterial adhesion depends not only on the ability to quickly find the mannose receptor but also on the property of the two domains to then separate, allowing the conformational switch that strengthens binding.

The entire 95 kDa type 1 fimbrial tip complex of *E. coli*, which includes three other structural proteins in addition to FimH, was first tested for the ability to support the shear dependent properties of FimH. Not only was the purified tip complex able to reproduce the shear-dependent binding to a mannose-coated surface demonstrated by fimbriated bacteria, but the force-enhanced interaction between the tip-associated FimH and mannose could be shown in the single molecule force spectroscopy experiments in which force is increased at a constant loading rate with an atomic force microscope. Thus, the shear-dependent and force-enhanced binding properties are intrinsic to the fimbrial tip complex crystallized recently [8].

**Figure 7:**
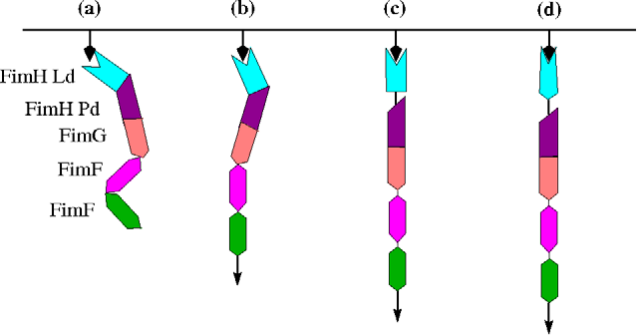
Cartoon illustrating force-induced conformational changes in fimbrial tips. The mannose substrate is represented by black arrows attached to a surface, whereas tensile force is indicated by arrows attached to the second copy of FimF. (a) Fimbrial tip initially binds to mannose. (b) Tensile force due to flow straightens the flexible FimF interfaces. (c) Transition state where FimH Ld and Pd are separated but Ld is still in the low-affinity state. (d) Final conformation under flow with FimH Ld in the high-affinity state [8].

Currently, it is very difficult to evaluate the quaternary structural properties of such a large protein complex like the fimbrial tip under dynamic conditions by using direct experimental approaches like NMR or other forms of protein spectroscopy analysis. Though some predictions were made from the analysis of the static crystallographic structure, here we used molecular dynamics simulations as a tool to study in silico the dynamic properties of complex biomolecular structures in solution. It would have been more realistic to perform the simulations with mannose in the binding pocket and apply the force to mannose instead of the 13 mannose-interacting residues. However, the crystallographic structure of the fimbrial tip with FimH in the low-affinity state (where Ld and Pd are interacting with each other) does not contain mannose. Furthermore, a conformational switch of Ld to the high-affinity state (where the domains are separated) is not expected to occur in the time scale of the simulations. Thus, applying the force onto mannose would have had little influence onto the outcome of the simulations, where the goal was mainly the description of how Ld and Pd separate. The key findings of the simulations with and without force are summarized by a cartoon presented in Figure 7a–d.

Before tensile force is applied, Ld and Pd are docked onto each other forming a hook shaped structure (Figure 7a) stabilized by inter-domain side chain interactions and hydrogen bonds. In contrast, the interface between FimG and FimF presents very few stabilizing contacts and thus is flexible. This interface, and most likely FimF-FimF as well, acts like a hinge allowing for exploration and fast on rates. Moreover, upon application of tensile force, FimG and FimF separate from each other (Figure 7b) while their rotational freedom is still preserved (Figure 4e). This flexibility despite the presence of force is likely to reduce the torsional forces on mannose in the binding pocket, stabilizing the adhesive interaction under dynamic flow conditions.

Importantly, upon further application of tensile force, most of the interactions between Ld and Pd also break and the hook shaped structure of FimH straightens with the domains separating (Figures 3g and 7c). As discussed above, separation of the domains leads to a switch of Ld from a low- to a high-affinity state (Figure 7d) [16],[35]. Thus, while the hinge opening is the primary event in the native fimbrial tip-incorporated FimH that is required for the catch bond to be formed, it is the ensuing conformational change within the binding domain itself that is a key event in strengthening the bond with mannose, observed in single molecule force spectroscopy here and in previous studies [8],[17]. Notably, despite the fact that, in the absence of force, the Ld-Pd interface is stabilized by more bonds and is more stable than the interface between Pd and FimG, Ld separates from Pd when force is applied, whereas the Pd-FimG interface remains almost unaltered. Thus, we propose here that the hooked conformation of FimH is of key significance because it allows for fast sequential unzipping of the contacts stabilizing the Ld-Pd interface. At the same time, the rigid open Pd-FimG conformation provides a lever arm that facilitates the FimH hook opening under force (Figure 7c).

In summary, every interface in the fimbrial tip appears to play a specific functional role. The FimG-FimF (and likely also FimF-FimF) interface presents lateral and rotational flexibility to efficiently explore the target surface and prevent the stress applied through the fimbrial rod from disrupting the interaction between FimH and the receptor. The hook shaped structure of FimH acts like a force sensor that is activated if a force threshold is reached. Finally, the relatively stiff interface between Pd and FimG extends the lever arm of the pilin domain. This may explain why so many different subunits are found in the fimbrial tip, instead of FimH being directly attached to the major subunit, FimA. The function of the fimbrial tip can be compared to that of an anchor connected to a flexible chain. The flexible connection to the chain allows the relatively stiffer hook to efficiently explore the surrounding tissue until it engages in bond formation. After the bond is formed, the mechanical stress onto the bacterial cell is transmitted to the hook, which acts as a force sensor switching to a high-affinity state.

The importance of mechanical forces in modulating biological adhesion is highlighted by the structure and function of the adhesive fimbrial tip described here. In particular, the quaternary conformation of this adhesive complex is optimized for initializing the binding (fast on rates), fast switching to the strong bond, and sustaining the binding under dynamic shear conditions (slow off rates). It needs to be noted that the rest of the fimbrial rod is also likely to play an important role in shear conditions. Namely, it has been shown that the fimbrial rod can uncoil and prevent breakage of the high-affinity mannose bond when the force load becomes too high [36]. The rod can recoil when external force drops, sustaining internal mechanical tension on FimH to keep it in a high-affinity conformation by preventing re-docking of the two domains into the hook conformation.

The type 1 fimbrial tip is the only complex of a native fimbrial tip described at atomic resolution so far. It is possible that the quaternary conformation of adhesive tips in other fimbriae will have similar mechanical properties. For example, di-galactose-specific P fimbriae of *E. coli* also exhibit shear-enhanced binding [28] and possess a complex filamentous adhesive tip. The multi-domain structures of some eukaryotic adhesins have similarities to the FimH fimbrial tip as well.

It has been shown that many other receptor-like interactions in eukaryotes are also governed by the catch-bond mechanism. Examples include the binding mechanism of selectins [20],[37],[38], integrins [39], and also the interaction between von Willebrand Factor and the platelet surface receptor glycoprotein Ibα [40],[41], between actin and myosin [42], and between kinetochores and single microtubules [43]. The distinctive hinge or hook shape between the adhesive and anchoring domains of the P-/L-selectins [37],[44], integrins [45],[46], and myosin [47], as well as the curled shape of microtubule protofilaments in the weak-binding disassembly mode [48], suggest a similar mechanism by which mechanical force can apply enough energy to activate these proteins and complexes [19],[46],[49]. Indeed, a common characteristic of protein complexes displaying catch bond behavior is the presence of multiple domains or subunits. One role for the many nonadhesive domains in integrins, and the two to nine consensus repeats in selectins, for example, might be to confer additional molecular flexibility to enhance binding rates and minimize torsional forces. The similarities in quaternary structural mechanics between type 1 fimbrial tips in bacteria and these eukaryotic structures suggest convergent evolution, in which a wide range of proteins that must mediate adhesion in dynamic conditions have evolved multiple domains or subunits creating both rigid hooks to capture energy from mechanical force to initiate conformational changes and flexible regions to initiate and stabilize adhesion in dynamic conditions. This raises the question as to whether the quaternary structures of other multidomain adhesive complexes, including matrix proteins like fibronectin, blood proteins like fibrinogen and von Willebrand Factor, cell-cell adhesion proteins like cadherins, and receptors like selectins and platelet GPIbα, are also optimized for mechanical functions. As novel native structures of adhesive complexes are elucidated in the future, the paradigm of mechanically regulated cell adhesion will provide new insights into the molecular details and physiological conditions of cell-cell interactions.

It is possible that quaternary structural changes are also involved in other biological interactions that are exposed to mechanical force, even if they do not involve catch bonds. Force regulation and, thus, the catch-bond mechanism might not provide advantages in interactions that provide irreversible adhesion, like between the holdfast of bacteria and colonizing surface that involves one of the strongest adhesive biological interactions [50]. Catch bonds are also unlikely to be involved in the interactions where the binding strength is regulated by the chemical modification of the receptor or ligand, like covalent chemistry performed by adhering mussels [51]. At the same time, other adhesive interactions that provide reversible adhesion, like the foot of the gecko where spatula use pre-tension to control the adhesion strength that drops off at a critical angle, [52],[53] could benefit from some version of force regulation of the quaternary structures of the involved proteins. It remains to be determined, however, to what extent the catch-bond mechanism can be generalized to any reversible (non-covalent) biological interactions regulated by mechanical force.

## Materials and Methods

### Flow Chamber Experiments

#### Bacterial binding to uroepithelial cells

Parallel plate flow chamber experiments were in general performed as described previously [5]. A confluent monolayer of T24 human urinary bladder carcinoma cells was grown in Corning brand polystyrene tissue culture dish in McCoy medium supplemented with 10% FBS and penicillin/streptomycin. Flow chamber with 1 cm wide gasket was assembled on top of the monolayer. Recombinant GFP-expressing *E. coli* were grown overnight in LB with shaking, washed, and resuspended in McCoy medium to OD 1.0. Then *E. coli* were flown into the chamber at low (0.01 Pa) or high (0.1 Pa) shear stress conditions for 30 min at 37°C. Then the flow was switched to McCoy medium without bacteria, and the flow chamber was mounted on a Nikon TE200 inverted microscope with a 10-fold phase-contrast objective and fluorescent lamp. The T24 cells monolayer was examined under transmitted light, and images of several fields of view per each plate with intact monolayer were acquired both in transmitted and fluorescent green light. The number of green dots, which corresponds to bacteria sticking on the surface, was counted and averaged over 10 pictures at each shear. Examples of these pictures for both low and high shear are presented in Figure 2a.

#### Bacterial binding to mannose

Binding of bacteria to mannose coated dishes was performed as described previously [32]. The dishes coated with monomannosylated bovine serum albumin were inserted into a parallel plate flow chamber. Then *E. coli* were washed into the chamber at either 0.01 Pa or 0.1 Pa for 5 min at 37°C and the number of bacteria sticking onto the surface was determined as described in “Bacterial Binding to Uroepithelial Cells.” A comparison of *E. coli* binding to T24 cells or mannose at both shears is presented in Figure 2b.

#### Fimbrial tip binding to mannose

Polystyrene beads with a 3 µm diameter were incubated with 100 µg/ml mannosylated bovine serum albumin (V-labs Inc., Covington, LA) for 75 min and washed with 0.2% bovine serum albumin in phosphate buffered saline (PBS-BSA) to reduce nonspecific binding. Fimbrial tips were immobilized on a Corning brand polystyrene tissue culture dish at 0.1 µg/ml total protein for 1.25 h at 37°C, then blocked with PBS-BSA overnight. To measure the rate of accumulated binding of beads to the surface, the suspended beads were washed over the surface at the indicated shear stress levels for 5 min and the number of adherent beads at the end of 5 min was counted and plotted in Figure 2c.

### Constant Velocity Single Molecule Force Spectroscopy

Single molecule force spectroscopy experiments were performed as described previously [7]. Fimbrial tips were immobilized on plates as described above except at a much higher concentration on account of the small size of the atomic force microscope (AFM) cantilever tip. Olympus Biolever cantilevers were incubated with 100 µg/ml man-BSA at 37°C for 1.25 h and blocked overnight in PBS-BSA. An Asylum MFP-3D AFM was used to probe the forces on single bonds between the cantilever and surface in PBS-BSA. The tip was pressed to the surface and then the tip withdrawn at a constant velocity calculated to create 300 pN/s, given the spring constant of the cantilever tips (calculated with the thermal method and found to vary between 4.38 and 5.69 pN/nm for different tips). The force at rupture was calculated as the difference between the peak of tensile force and the average baseline force following rupture, using an automated script. Nonspecific interactions between the tip and surface were measured by adding 4% alpha-methyl mannose to the PBS-BSA solution to prevent specific bonds from forming. The resulting histogram of rupture forces was plotted as a difference between the number of total events and non-specific rupture events at a given force range (Figure 2d, bars). The off rates (Figure 2d, circles) were then calculated as a function of force according to the method of Evans et al. [18]. The probability of rupture was estimated for the *k* ^th^ force bin by dividing the number of interactions that break in the force range represented by the *k* ^th^ bin (Δ*N_k_*) by the number remaining at the start of the bin (*N_k_* = Δ*N_k_*+Δ*N_k_*_+1_+…). The rate of rupture was calculated as this probability divided by Δ*t_k_*, the time spent in that bin, calculated as the width of force bin divided by loading rate.

### Initial Structures and Definition of Domains

The conformations used in the molecular dynamics simulations were derived from the crystallographic structure of the fimbrial tip (PDB code 3JWN). The quaternary structure of the crystallized fimbrial tip consists of four subunits and a chaperone protein FimC (Figure 1a). Each subunit donates a β strand to the N-terminal neighboring subunit, thus allowing for polymerization (Figure 1b). In order to study the flexibility and the function of the fimbrial tip, the entire structure was subdivided into domains. Each domain consisted of the globular part of a subunit and included the strand donated by the neighboring subunit. The donated strand is covalently linked to the neighboring domain through a short unstructured chain, termed a linker chain. Starting from the N-terminus, the names and in parenthesis the exact amino acid sequences of each domain are as follows: FimH lectin (1–158), FimH pilin (161–279 and 1–12 of donated strand by FimG subunit), FimG (16–144 and 1–12 of donated strand by FimF subunit), and FimF (16–154 and 1–12 of donated strand by the second FimF). In this article, if not specified, FimG and FimF always refer to the domains and FimH to the lectin domain (Ld) and pilin domain (Pd) including the linker chain. Otherwise, the word “subunit” will accompany the name, e.g., FimG sub-unit.

The constant force simulations (see section “Simulations”) were run with the first four domains of the fimbrial tip, i.e., FimH Ld, FimH Pd, FimG, and FimF, after having been equilibrated for 3 ns. In this construct, the strand donated by the second copy of FimF was truncated after residue 14. In the simulations run with just FimH, the protein was cleaved after residue 13 of the FimG donated strand. In the runs with FimH Pd-FimG or FimG-FimF, the protein was cleaved after residue 14 of the respective donated strand.

### Simulations

The MD simulations were performed with the program NAMD [54] using the AMBER03 force field [55] and the TIP3P model of water [56]. In the simulations with only two domains, the protein was inserted into a cubic water box with a side length of 115 Å such that the distance between the protein and the edge of the box measured at least 12 Å. In the simulations with the four domains (FimH Ld to FimF), a rectangular water box was used with side lengths of 70 Å×70 Å×200 Å to allow for extension of the protein when a pulling force was applied. The water molecules overlapping with the protein or the ions were removed if the distance between the oxygen atom of a water molecule and any atom of the protein or any ion was smaller than 3.1 Å. This value is equivalent to the distance between water molecules at room temperature and 1 atmosphere of pressure. To avoid finite size effects, periodic boundary conditions were applied. Different initial random velocities were assigned whenever more than one simulation was performed with the same molecule. Electrostatic interactions were calculated within a cutoff of 10 Å, while long-range electrostatic effects were taken into account by the Particle Mesh Ewald summation method [57]. Van der Waals interactions were truncated with the use of a switch function starting at 8 Å and turning off at 10 Å.

Before production, the starting conformation and the solvent were minimized by performing 6,000 steps of the conjugate gradient method. Following minimization, the system was heated by increasing the temperature stepwise in increments of 10 K each every 1 ps during a total time of 30 ps until the target temperature of 300 K was reached. During production, the temperature was kept constant by using the Berendsen thermostat [58] with a relaxation time of 0.1 ps, while the pressure was held constant at 1 atm by applying a pressure piston [59]. For the 300 K runs where no external force was applied, the first 10 ns of unconstrained simulation time were also considered part of the equilibration and were not used for the analysis. The dynamics were integrated with a time step of 2 fs. The covalent bonds involving hydrogens were rigidly constrained by means of the SHAKE algorithm with a tolerance of 10^−8^. Snapshots were saved every 10 ps for trajectory analysis.

The simulations where no external force was applied were checked for convergence by calculating averages and standard deviations of the magnitudes presented in Figure 4 on time windows of 10 ns. In the case of the simulations with either the entire FimH protein or the complex between Pd and FimG, the averages over the 10 ns time window differed on values smaller than the standard deviations, indicating that the runs had converged. On the other hand, the quaternary structure of the FimG-FimF complex was observed to be highly flexible because of the near absence of inter-protein contacts; thus, the averages over 10 ns time windows had relatively larger fluctuations.

#### Constant force pulling

A force of 200 pN was applied in opposite directions to simulate the extension of the four domains (FimH Ld to FimF). The force was applied to the Cα of residue 14 of the FimF donated strand located at the C-terminus and to the center of mass of the Cα atoms of the following 13 residues located near the mannose binding pocket at the N-terminus: F1, I13, N46, D47, Y48, I52, D54, Q133, N135, Y137, N138, D140, and D141. These are the same residues used to pull the protein in a previous study by us [5]. Though the fimbrial tip crystal structure was obtained in the absence of mannose, an assumption was made that these residues interact with mannose based on the previously obtained crystal structure of the FimH-FimC complex with separated FimH domains [9],[11]. The force was applied during in total 10 ns and three runs were performed. Prior to pulling, the four-domain construct was equilibrated at 300 K for 3 ns. In addition, three 10 ns pulling simulations were performed also with the isolated FimH protein, where the initial structure was taken after 10 ns equilibration of the 40 ns run at 300 K (Table 2).

### Determination of Native Contacts

The conformations sampled at room temperature were used to determine native hydrogen bonds and salt bridges. To define a hydrogen bond, a H … O distance cutoff of 2.7 Å and a D–H … O angle cutoff of 120° was used, where a donor D could either be an oxygen or a nitrogen. Side chains were defined to form a contact when the distance between their geometric centers was not larger than 6 A. An interaction was defined as salt bridge if the atoms Nζ of Lys or Cζ of Arg were closer than 4 Å or 5 Å, respectively, from either the Cγ of Asp or Cδ of Glu. All histidines were assumed neutral. Those hydrogen bonds and side chain contacts present in at least 66% of the frames of the 300 K simulations were selected as native contacts. Native inter-domain contacts were used to monitor the separation of the domains from each other in the pulling simulations.

### Inter-Domain Buried Surface and Angles

#### Inter-domain buried surface

The solvent accessible surface area (SASA) buried at the interface between two domains was calculated by subtracting the SASA of the two domains without the linker chain from the sum of the SASA of the two domains independently.

#### Inter-domain hinge and twist angles

The principal axes for each domain were calculated by diagonalizing the moment of inertia tensor using the program VMD [60]. The hinge angle between two neighboring domains was defined as the angle between their longest principal axes subtracted from 180°. The twist angle was defined as the angle between the second longest principal axes.

### Calculation of the Elongation of the Protein

Elongation of the fimbrial tip under pulling was calculated between the N-terminus of FimH (residue 1) and the C-terminus of FimG (residue Asp12 of the donated FimF strand) because the interface between FimG and FimF is rather flexible whereas the interface between Pd and FimG is relatively stiff (Figure 4). This distance for the native state was determined by averaging over the last 2 ns of a 3 ns simulation with the entire fimbrial tip, where no force was applied, and measured 121±1 Å. Because of the high force used in the pulling simulations, the single domains were likely to experience overstretching. Thus stretching of the single domains was subtracted by calculating the distance between the termini of the Ld, Pd, and FimG domains (see section “Initial Structures and Definition of Domains” above for the exact definition of domains) and comparing it to their average lengths during the last 2 ns of the 3 ns run with the fimbrial tip.

## Supporting Information

Figure S1 Comparison between different conformations of fimbrial tip domains and principal axes. (a) Crystallographic structure of the fimbrial tip containing the four domains used in the simulations. The angle between the first principal components of neighboring domains (hinge angle) is indicated. (b) Conformation of FimH after 14 ns of simulation at 300 K from the 40 ns run. (c) Conformation of the complex between FimH pilin domain and FimG obtained after 14 ns of simulation at 300 K. (d) Conformation of the complex between FimG and FimF obtained after 14 ns of simulation at 300 K. Note that after 14 ns FimF is in a different configuration as in the X-ray structure. (e) Conformation of the fimbrial tip after pulling at constant force for 10 ns (pull 1).
(10.04 MB EPS)

Figure S2 Time series of quantities describing the flexibility of domains during room temperature simulations (of the three 300 K simulations with FimH, the longest, 40 ns, is shown here). (a) Cα RMSD of adjacent domains from the native structure. (b) Cα RMSD of single domains from the native structure. (c) Distance between the centers of mass of adjacent domains. (d) Surface buried at the interface between adjacent domains. (e) Hinge and (f) twist angle between adjacent domains (see also Figure 1 and “Materials and Methods”). The twist angle between FimH Pd and FimG is negative and the corresponding ordinate is indicated on the right. (g) Number of native side-chain contacts between adjacent domains. The native side-chain contacts were determined from 300 K runs performed with pairwise domains (see “Materials and Methods”).
(1.16 MB EPS)

Figure S3 Time series of quantities describing conformational changes at the interface between domains during the pull 1 simulation. Prior to pulling, 3 ns of equilibration were performed and are shown in the left part of the plot. All plotted quantities are time averages over a window of 200 ps. (a) Pairwise Cα RMSD of adjacent domains from the native structure. (b) Distance between the centers of mass of adjacent domains. (c) Surface buried at the interface between adjacent domains. (d) Hinge and (e) twist angle between adjacent domains (see also Figure 1 and “Materials and Methods”). The twist angle between FimH Pd and FimG is negative and the corresponding ordinate is indicated on the right. (f) Number of native side-chain contacts between adjacent domains. The native side-chain contacts were determined from 300 K runs performed with pairwise domains (see “Materials and Methods”). (g) Solvent accessible surface area of the carboxyl oxygen of S114 and the amid group of C161, which are involved in a native inter-domain hydrogen bond.
(0.39 MB EPS)

Figure S4 Time series of quantities describing conformational changes at the interface between domains during the pull 2 simulation. Prior to pulling, 3 ns of equilibration were performed and are shown in the left part of the plot. All plotted quantities are time averages over a window of 200 ps. (a) Pairwise Cα RMSD of adjacent domains from the native structure. (b) Distance between the centers of mass of adjacent domains. (c) Surface buried at the interface between adjacent domains. (d) Hinge and (e) twist angle between adjacent domains (see also Figure 1 and “Materials and Methods”). The twist angle between FimH Pd and FimG is negative and the corresponding ordinate is indicated on the right. (f) Number of native side-chain contacts between adjacent domains. The native side-chain contacts were determined from 300-K runs performed with pairwise domains (see “Materials and methods”). (g) Solvent accessible surface area of the carboxyl oxygen of S114 and the amid group of C161 which are involved in a native inter-domain hydrogen bond.
(0.39 MB EPS)

Figure S5 Time series of quantities describing conformational changes at the interface between domains during the pull 3 simulation. Prior to pulling, 3 ns of equilibration were performed and are shown in the left part of the plot. All plotted quantities are time averages over a window of 200 ps. (a) Pairwise Cα RMSD of adjacent domains from the native structure. (b) Distance between the centers of mass of adjacent domains. (c) Surface buried at the interface between adjacent domains. (d) Hinge and (e) twist angle between adjacent domains (see also Figure 1 and “Materials and Methods”). The twist angle between FimH Pd and FimG is negative and the corresponding ordinate is indicated on the right. (f) Number of native side-chain contacts between adjacent domains. The native side-chain contacts were determined from 300 K runs performed with pairwise domains (see “Materials and Methods”). (g) Solvent accessible surface area of the carboxyl oxygen of S114 and the amid group of C161, which are involved in a native inter-domain hydrogen bond.
(0.39 MB EPS)

Figure S6 Time series of the formation of native inter-domains contacts during the pull 1 simulation. The time series during the 3 ns equilibration run prior to start pulling are displayed on the left and separated from the pulling plots by a vertical dashed line. Side chain contacts between FimH Ld and FimH Pd are colored in cyan, those between FimH Pd and FimG in orange, and those between FimG and FimF in magenta. Native inter-domains hydrogen bonds, observed only between FimH Ld and FimH Pd, are colored in blue. The ordinate on the left lists the amino acids involved in contacts. The residue on the left of the “-” is contained in the domain closer to the N-terminus. In most cases, residues involved in inter-domain contacts are also contained within the subunit with the same name. The only exception is residue R12 in the FimF subunit, which belongs to the FimG domain and contacts A59 in FimF (see “Materials and Methods” for the definition of sub-domains and subunits).
(1.07 MB EPS)

Figure S7 Time series of the formation of native inter-domains contacts during the pull 2 simulation. The time series during the 3 ns equilibration run prior to start pulling are displayed on the left and separated from the pulling plots by a vertical dashed line. Side chain contacts between FimH Ld and FimH Pd are colored in cyan, those between FimH Pd and FimG in orange, and those between FimG and FimF in magenta. Native inter-domains hydrogen bonds, observed only between FimH Ld and FimH Pd, are colored in blue. The ordinate on the left lists the amino acids involved in contacts. The residue on the left of the “-” is contained in the domain closer to the N-terminus. In most cases, residues involved in inter-domain contacts are also contained within the subunit with the same name. The only exception is residue R12 in the FimF subunit, which belongs to the FimG domain and contacts A59 in FimF (see “Materials and Methods” for the definition of sub-domains and subunits).
(1.05 MB EPS)

Figure S8 Time series of the formation of native inter-domains contacts during the pull 3 simulation. The time series during the 3 ns equilibration run prior to start pulling are displayed on the left and separated from the pulling plots by a vertical dashed line. Side chain contacts between FimH Ld and FimH Pd are colored in cyan, those between FimH Pd and FimG in orange, and those between FimG and FimF in magenta. Native inter-domain hydrogen bonds, observed only between FimH Ld and FimH Pd, are colored in blue. The ordinate on the left lists the amino acids involved in contacts. The residue on the left of the “-” is contained in the domain closer to the N-terminus. In most cases, residues involved in inter-domain contacts are also contained within the subunit with the same name. The only exception is residue R12 in the FimF subunit, which belongs to the FimG domain and contacts A59 in FimF (see “Materials and Methods” for the definition of sub-domains and subunits).
(1.10 MB EPS)

Figure S9 Time series of the formation of native inter-domain contacts during all three pulling simulations with FimH. Side chain contacts between FimH Ld and FimH Pd are colored in cyan. Native inter-domain hydrogen bonds between FimH Ld and FimH Pd are colored in blue. The ordinate on the left lists the amino acids involved in contacts. The residue on the left of the “-” is contained in the domain closer to the N-terminus (see “Materials and Methods” for the definition of sub-domains and subunits and native contacts).
(2.44 MB EPS)

Figure S10 Cα RMSD at time of rupture versus distance to the hinge axis of residues involved in inter-domain contacts between Ld and Pd. The values were averaged over three pulling simulations with the fimbrial tip (left) and three pulling runs with the isolated FimH protein (right). A straight line was fitted to show that the contacts break in a sequential manner with contacts located farther away from the hinge axis (Figure 5a in the article) breaking earlier in the simulations. The Pearson's linear correlation coefficient is 0.70 and 0.88 for the runs with the fimbrial tip and the runs with isolated FimH, respectively, while the *p* values are 0.02 and 8e^−4^, respectively. Figure 5 in the article contains a ranking analysis of the Cα RMSDs versus distance from the axis averaged over all six pulling simulations.
(0.03 MB EPS)

Table S1 Estimate of the minimal amount of elongation that the FimH-FimG complex has to undergo until the transition state is reached (see “Materials and Methods” for details). The time point along the three pulling simulations is determined where the side chain of Arg166 loses its inter-domain contacts with Ld (one side chain contact with Val155 and two hydrogen bonds with the carbonyl oxygen of Ala115; Figures S6, S7, S8, S9). This event is observed to always be the first rupture event in all three simulations. Thus it is assumed that the location of the transition state will either be at exactly this event or later. It is worth mentioning that in three pulling simulations with just FimH, rupture of contacts involving Arg166 was also observed to be the first rupture event (Figures S9 and S10), providing further statistical evidence.
(0.03 MB DOC)

## Abbreviations

AFM: atomic force microscope
BSA: bovine-serum-albumin
Ld: lectin domain
MD: molecular dynamics
Pd: pilin domain
RMSD: root mean square deviation
SASA: solvent accessible surface area

## References

[1] Beachey E (1981). Bacterial adherence–adhesion-receptor interactions mediating the attachment of bacteria to mucosal surfaces.. J Infect Dis.

[2] Klemm P (1997). Fimbriae: adhesion, genetics, biogenesis, and vaccines.

[3] Hahn E, Wild P, Hermanns U, Sebbel P, Glockshuber R (2002). Exploring the 3D molecular architecture of Escherichia coli type 1 pili.. J Mol Biol.

[4] Jones C, Pinkner J, Roth R, Heuser J, Nicholes A (1995). FimH adhesin of type-1 pili is assembled into a fibrillar tip structure in the enterobacteriaceae.. Proc Natl Acad Sci U S A.

[5] Thomas W. E, Trintchina E, Forero M, Vogel V, Sokurenko E. V (2002). Bacterial adhesion to target cells enhanced by shear force.. Cell.

[6] Anderson B. N, Ding A. M, Nilsson L. M, Kusuma K, Tchesnokova V (2007). Weak rolling adhesion enhances bacterial surface colonization.. J Bacteriol.

[7] Yakovenko O, Sharma S, Forero M, Tchesnokova V, Aprikian P (2008). FimH forms catch bonds that are enhanced by mechanical force due to allosteric regulation.. J Biol Chem.

[8] Le Trong S. N, Aprikian P, Kidd B. A, Forero-Shelton M, Tchesnokova V (2010). Structural basis for mechanical force regulation of the adhesin FimH via finger trap-like β sheet twisting.. Cell.

[9] Choudhury D, Thompson A, Stojanoff V, Langermann S, Pinkner J (1999). X-ray structure of the FimC-FimH chaperone-adhesin complex from uropathogenic Escherichia coli.. Science.

[10] Gossert A. D, Bettendorff P, Puorger C, Vetsch M, Herrmann T (2008). NMR structure of the Escherichia coli type 1 pilus subunit FimF and its interactions with other pilus subunits.. J Mol Biol.

[11] Hung C, Bouckaert J, Hung D, Pinkner J, Widberg C (2002). Structural basis of tropism of Escherichia coli to the bladder during urinary tract infection.. Mol Microbiol.

[12] Eidam O, Dworkowski F. S. N, Glockshuber R, Gruetter M. G, Capitani G (2008). Crystal structure of the ternary FimC-FimF(t)-FimD(N) complex indicates conserved pilus chaperone-subunit complex recognition by the usher FimD.. FEBS Lett.

[13] Puorger C, Eidam O, Capitani G, Erilov D, Gruetter M. G (2008). Infinite kinetic stability against dissociation of supramolecular protein complexes through donor strand complementation.. Structure.

[14] Bouckaert J, Berglund J, Schembri M, De Genst E, Cools L (2005). Receptor binding studies disclose a novel class of high-affinity inhibitors of the Escherichia coli FimH adhesin.. Mol Microbiol.

[15] Weissman S, Chattopadhyay S, Aprikian P, Obata-Yasuoka M, Yarova-Yarovaya Y (2006). Clonal analysis reveals high rate of structural mutations in fimbrial adhesins of extraintestinal pathogenic Escherichia coli.. Mol Microbiol.

[16] Thomas W, Forero M, Yakovenko O, Nilsson L, Vicini P (2006). Catch-bond model derived from allostery explains force-activated bacterial adhesion.. Biophys J.

[17] Tchesnokova V, Aprikian P, Yakovenko O, Larock C, Kidd B (2008). Integrin-like allosteric properties of the catch bond-forming FimH adhesin of Escherichia coli.. J Biol Chem.

[18] Kinoshita K, Leung A, Simon S, Evans E (2010). Long-lived, high-strength states of ICAM-1 bonds to beta(2) integrin, II: lifetimes of LFA-1 bonds under force in leukocyte signaling.. Biophys J.

[19] Thomas W. E, Vogel V, Sokurenko E (2008). Biophysics of catch bonds.. Annu Rev Biophys.

[20] Marshall B, Long M, Piper J, Yago T, McEver R (2003). Direct observation of catch bonds involving cell-adhesion molecules.. Nature.

[21] Le Trong I, Aprikian P, Kidd B. A, Thomas W. E, Sokurenko E. V (2010). Donor strand exchange and conformational changes during *E. coli* fimbrial formation.. J Struct Biol.

[22] Evans E (2001). Probing the relation between force - lifetime - and chemistry in single molecular bonds.. Ann Rev Biophys Biomol Struct.

[23] Kramers H (1940). Brownian motion in a field of force and the diffusion model of chemical reactions.. Physica.

[24] Izrailev S, Stepaniants S, Balsera M, Oono Y, Schulten K (1997). Molecular dynamics study of unbinding of the avidin-biotin complex.. Biophys J.

[25] Isralewitz B, Baudry J, Gullingsrud J, Kosztin D, Schulten K (2001). Steered molecular dynamics investigations of protein function.. J Mol Graph Model.

[26] Nilsson L. M, Thomas W. E, Sokurenko E. V, Vogel V (2008). Beyond induced-fit receptor-ligand interactions: structural changes that can significantly extend bond lifetimes.. Structure.

[27] Whitfield M, Ghose T, Thomas W (2010). Shear-stabilized rolling behavior of E. coli examined with simulations.. Biophys J.

[28] Nilsson L, Thornas W, Sokurenko E, Vogel V (2006). Elevated shear stress protects Escherichia coli cells adhering to surfaces via catch bonds from detachment by soluble inhibitors.. Appl Environ Microbiol.

[29] Aprikian P, Tchesnokova V, Kidd B, Yakovenko O, Yarov-Yarovoy V (2007). Interdomain interaction in the FimH adhesin of Escherichia coli regulates the affinity to mannose.. J Biol Chem.

[30] Sokurenko E, Chesnokova V, Doyle R, Hasty D (1997). Diversity of the Escherichia coli type 1 fimbrial lectin—differential binding to mannosides and uroepithelial cells.. J Biol Chem.

[31] Weissman S. J, Beskhlebnaya V, Chesnokova V, Chattopadhyay S, Stamm W. E (2009). Differential stability and trade-off effects of pathoadaptive mutations in the Escherichia coli FimH adhesin (vol. 75, pg 3548, 2007).. Infection and Immunity.

[32] Thomas W. E, Nilsson L. M, Forero M, Sokurenko E. V, Vogel V (2004). Shear-dependent ‘stick-and-roll’ adhesion of type 1 fimbriated Escherichia coli.. Mol Microbiol.

[33] Jeffrey B, Udaykumar H, Schulze K (2003). Flow fields generated by peristaltic reflex in isolated guinea pig ileum: impact of contraction depth and shoulders.. Am J Physiol Gastrointest Liver Physiol.

[34] Lentle R. G, Janssen P. W. M (2008). Physical characteristics of digesta and their influence on flow and mixing in the mammalian intestine: a review.. J Comp Physiol B Biochem Syst Environ Physiol.

[35] Thomas W (2008). Catch bonds in adhesion.. Annu Rev Biomed Eng.

[36] Forero M, Yakovenko O, Sokurenko E. V, Thomas W. E, Vogel V (2006). Uncoiling mechanics of Escherichia coli type I fimbriae are optimized for catch bonds.. PLoS Biol.

[37] Phan U. T, Waldron T, Springer T. A (2006). Remodeling of the lectin-EGF-like domain interface in P- and L-selectin increases adhesiveness and shear resistance under hydrodynamic force.. Nat Immunol.

[38] Sarangapani K, Yago T, Klopocki A, Lawrence M, Fieger C (2004). Low force decelerates L-selectin dissociation from P-selectin glycoprotein ligand-1 and endoglycan.. J Biol Chem.

[39] Kong F, Garcia A. J, Mould A. P, Humphries M. J, Zhu C (2009). Demonstration of catch bonds between an integrin and its ligand.. J Cell Biol.

[40] Yago T, Lou J, Wu T, Yang J, Miner J. J (2008). Platelet glycoprotein Iba forms catch bonds with human WT vWF but not with type 2B von Willebrand disease vWF.. J Clin Invest.

[41] Interlandi G, Thomas W. E (2010). The catch bond mechanism between von Willebrand Factor and platelets investigated by molecular dynamics simulations.. Proteins: Struct, Funct, Bionf.

[42] Guo B, Guilford W. H (2006). Mechanics of actomyosin bonds in different nucleotide states are tuned to muscle contraction.. Proc Natl Acad Sci U S A.

[43] Uezumi A, Fukada S-I, Yamamoto N, Takeda S. i, Tsuchida K (2010). Mesenchymal progenitors distinct from satellite cells contribute to ectopic fat cell formation in skeletal muscle.. Nat Cell Biol.

[44] Lou J, Yago T, Klopocki A. G, Mehta P, Chen W (2006). Flow-enhanced adhesion regulated by a selectin interdomain hinge.. J Cell Biol.

[45] Xiong J, Stehle T, Diefenbach B, Zhang R, Dunker R (2001). Crystal structure of the extracellular segment of integrin alpha V beta 3.. Science.

[46] Astrof N. S, Salas A, Shimaoka M, Chen J, Springer T. A (2006). Importance of force linkage in mechanochemistry of adhesion receptors.. Biochemistry.

[47] Holmes K, Schroder R, Sweeney H, Houdusse A (2004). The structure of the rigor complex and its implications for the power stroke.. Philos Trans R Soc Lond B Biol Sci.

[48] Mandelkow E. M, Mandelkow E, Milligan R. A (1991). Microtubule dynamics and microtubule caps—a time-resolved cryoelectron microscopy study.. J Cell Biol.

[49] Franck A. D, Powers A. F, Gestaut D. R, Gonen T, Davis T. N (2007). Tension applied through the Dam1 complex promotes microtubule elongation providing a direct mechanism for length control in mitosis.. Nat Cell Biol.

[50] Tsang P. H, Li G. L, Brun Y. V, Ben Freund L, Tang J. X (2006). Adhesion of single bacterial cells in the micronewton range.. Proc Natl Acad Sci U S A.

[51] Lee H, Scherer N. F, Messersmith P. B (2006). Single-molecule mechanics of mussel adhesion.. Proc Natl Acad Sci U S A.

[52] Autumn K, Liang Y. A, Hsieh S. T, Zesch W, Chan W. P (2000). Adhesive force of a single gecko foot-hair.. Nature.

[53] Chen B, Wu P. D, Gao H. J (2009). Pre-tension generates strongly reversible adhesion of a spatula pad on substrate.. J R Soc Interface.

[54] Kalè LaS R, Bhandarkar M, Brunner R, Gursoy A, Krawetz N (1999). NAMD2: greater scalability for parallel molecular dynamics.. Journ Comp Physics.

[55] Duan Y, Wu C, Chowdhury S, Lee M. C, Xiong G (2003). A point-charge force field for molecular mechanics simulations of proteins based on condensed-phase quantum mechanical calculations.. J Comput Chem.

[56] Mahoney M. W, Jorgensen W. L (2000). A five-site model for liquid water and the reproduction of the density anomaly by rigid, nonpolarizable potential functions.. J Chem Phys.

[57] Darden T, York D, Pedersen L (1993). Particle mesh ewald—an N.log(N) method for ewald sums in large systems.. J Chem Phys.

[58] Berendsen H. J. C, Postma J. P. M, F. V. G. W, Dinola A, Haak J. R (1984). Molecular-dynamics with coupling to an external bath.. J Chem Phys.

[59] Feller S. E, Zhang Y. H, Pastor R. W, Brooks B. R (1995). Constant-pressure molecular-dynamics simulation—the Langevin piston method.. J chem phys.

[60] Humphrey W, Dalke A, Schulten K (1996). VMD: visual molecular dynamics.. J Molec Graphics.

[61] Hayward S, Berendsen H (1998). Systematic analysis of domain motions in proteins from conformational change: new results on citrate synthase and T4 lysozyme.. Proteins: Struct, Funct, Genet.

