# Supplementary material for: The Bacterial Fimbrial Tip Acts as a Mechanical Force Sensor
Source: PLoS Biol. 2011 May 10;9(5):e1000617. doi: 10.1371/journal.pbio.1000617 (PMC3091844; doi:10.1371/journal.pbio.1000617)
Supplement: Table S1 — Estimate of the minimal amount of elongation that the FimH-FimG complex has to undergo until the transition state is reached (see “Materials and Methods” for details). The time point along the three pulling simulations is determined where the side chain of Arg166 loses its inter-domain contacts with Ld (one side chain contact with Val155 and two hydrogen bonds with the carbonyl oxygen of Ala115; Figures S6, S7, S8, S9). This event is observed to always be the first rupture event in all three simulations. Thus it is assumed that the location of the transition state will either be at exactly this event or later. It is worth mentioning that in three pulling simulations with just FimH, rupture of contacts involving Arg166 was also observed to be the first rupture event (Figures S9 and S10), providing further statistical evidence. (0.03 MB DOC) [file pbio.1000617.s011.doc]

**TABLE S1**

| Simulation | FimH-FimG elongation |
| --- | --- |
| Pull_1 | 7.44 |
| Pull_2 | 10.47 |
| Pull_3 | 9.3 |
| Average | 9.07 (1.25) |
